# Patient-Reported Outcomes Following Minimally Invasive Versus Conventional Orthognathic Surgery

**DOI:** 10.3390/cmtr19040042

**Published:** 2026-10-15

**Authors:** Felix Jose Amarista, Hannah Grimes, Domenic Digioia, Edward Ellis

**Affiliations:** 1 Department of Oral and Maxillofacial Surgery, Medical University of South Carolina, Charleston, SC 29425, USA; 2 Department of Oral and Maxillofacial Surgery, University of Texas Health San Antonio, San Antonio, TX 78229, USA; grimesh@uthscsa.edu (H.G.); digioia@uthscsa.edu (D.D.); ellise3@uthscsa.edu (E.E.III)

**Keywords:** orthognathic surgery, minimally invasive surgery, patient-reported outcomes, quality of life, postoperative recovery, patient expectations

## Abstract

Minimally invasive orthognathic surgery (MIOS) aims to reduce surgical morbidity and improve postoperative recovery; however, its effect on patient-reported recovery and quality of life compared with conventional orthognathic surgery (COS) remains unclear. This prospective longitudinal cohort study included patients with dentofacial deformities undergoing bimaxillary orthognathic surgery between June 2025 and June 2026. Patient-reported outcomes were assessed using the Short-Term Expectations (STE), Postsurgical Perceptions (PSP), Orthognathic Quality of Life Questionnaire (OQLQ), and Oral Health Impact Profile-14 (OHIP-14). Longitudinal outcomes were evaluated using linear mixed-effects models. Forty-one patients were included (20 COS and 21 MIOS), with no losses to follow-up. PSP, OHIP-14, and OQLQ scores changed significantly over time (all *p* < 0.001), without significant differences in recovery trajectories between groups. The largest observed PSP difference occurred at 1 week (COS, 102.5 ± 30.1; MIOS, 84.5 ± 26.2; *p* = 0.082). Higher baseline STE scores were independently associated with higher postoperative PSP scores (β = 7.40; SE = 2.87; *p* = 0.014). Although differences between-group were not statistically significant, postoperative symptom scores were lower in the MIOS cohort throughout follow-up, with the greatest difference observed during early recovery. Larger studies are needed to determine whether these observed differences represent clinically meaningful patient-reported benefits of MIOS.

## 1. Introduction

Conventional orthognathic surgery (COS) has proven to be a reliable and predictable procedure; however, patient recovery is often challenging. Minimally invasive surgery has emerged as a prevailing trend across various surgical specialties, driven by the overarching objective of reducing procedure-related morbidity while enhancing both patient recovery and outcomes. Minimally invasive orthognathic surgery (MIOS) represents a more recent and rapidly evolving development that has been gaining significant popularity around the world [1,2]. This procedure entails smaller incisions and minimal tissue dissection to mitigate complications and expedite the healing process. Additionally, it has been shown to offer numerous advantages, including improvements in aesthetics and facial expression, enhanced lip mobility, reduction in unwanted nasal widening, refinement of the jawline, and a decreased risk of nerve injury [3].

Numerous studies have demonstrated substantial enhancements in quality of life among patients who undergo orthognathic surgery [4,5,6,7]. However, despite MIOS technically reducing procedure-related morbidity and enhancing patient recovery and outcomes, no studies have assessed whether there is a difference in patient perceptions and quality of life.

The purpose of this study is to compare patient perceptions and quality of life in patients with dentofacial deformities undergoing conventional orthognathic surgery versus minimally invasive orthognathic surgery. We hypothesize that patients undergoing MIOS should experience improved perception and quality of life, particularly during the first few weeks after surgery.

## 2. Materials and Methods

### 2.1. Study Design/Sample

A prospective, comparative, longitudinal cohort study was conducted to evaluate patient-reported recovery and oral health-related quality of life after conventional orthognathic surgery (COS) and minimally invasive orthognathic surgery (MIOS). Patients were treated at the Department of Oral and Maxillofacial Surgery, University of Texas Health San Antonio, between June 2025 and June 2026. The study was approved by the institutional review board at UT Health San Antonio (MOD00004532).

The inclusion criteria were as follows: (1) patients aged 14 years or older; (2) diagnosis of a dentofacial deformity; (3) indication for bimaxillary orthognathic surgery; and (4) willingness to complete the scheduled patient-reported outcome assessments throughout the follow-up period. All adult participants provided informed consent. For participants younger than 18 years, written informed consent was obtained from a parent or legal guardian. Patients were excluded if they had cognitive impairment precluding completion of the questionnaires, a craniofacial syndrome, cleft lip and/or palate, or a maxillofacial deformity secondary to trauma or another disease. Patients who declined to participate, or who did not provide valid data for any of the follow-up questionnaires, were also excluded.

### 2.2. Variables

The primary predictor variable was the surgical approach, classified as COS or MIOS. The surgical technique was selected by the treating surgeon based on standard practice; patients were not randomly assigned to a technique. Surgeon A performed all MIOS procedures, and Surgeon E performed all COS procedures.

The primary outcome variable was patient satisfaction and quality of life pre- and post-surgery. Patient perceptions and satisfaction were assessed using (1) Short-Term Expectations (STE) [8] for the presurgical expectations; (2) Postsurgical Perceptions (PSP) [9] to assess the perceptions about the recovery process; oral health-related quality of life was assessed using the Orthognathic Quality of Life Questionnaire (OQLQ) [10,11] and the (4) Oral Health Impact Profile-14 (OHIP-14) [12].

The STE [8] assessed the discomfort or difficulty expected during the first month after surgery. Each item was rated using a 7-point scale from “expect no discomfort (1)” to “expect much discomfort (7).” A higher score indicates a higher expectation of problem or discomfort.

The PSP [9] assessed the recovery process and the level of current discomfort/difficulty. The PSP contains all the items on the STE as well as an additional 5 items in the dentofacial appearance subscale. PSP is based on 26 items that measure the postsurgical level of discomfort or difficulty experienced in the past month on a 7-point scale ranging from “none (1)” to “moderate/a lot of discomfort (7)”. A higher score is related to more struggles experienced by the patient.

The OQLQ [10,11] evaluated the general and facial appearance of patients undergoing orthognathic surgery. OQLQ comprises 22 items qualified from 1 to 4 points, in which 1 point means “it bothers you a little” while 4 points mean “it bothers you a lot”. The twenty-two items are divided into components: the first component is related to the social relationships of deformity, the second component is related to facial aesthetics, the third component is to oral function, and the fourth component is to awareness of dentofacial deformity. The total score ranges from 0 to 88. Higher scores mean lower quality of life, while lower scores mean higher quality of life.

The OHIP-14 [12] is a scale that assesses functional limitation, physical pain, psychosocial discomfort, physical disability, psychosocial disability, social disability, and handicap reported by patients concerning their oral conditions. It evaluates patients’ perception of the social effects of their oral health conditions using a 0 to 4 Likert scale. High values reflect the patients’ important affection, and low scores reflect that they are in a good mental state.

The covariates were analyzed as a continuous variable. The dentofacial diagnosis was classified into 3 categories: Class II, Class III, and other or not classifiable conditions. The model evaluating the association between baseline STE and postoperative PSP scores was adjusted for age, surgical group, time, and the group × time interaction, without inclusion of dentofacial diagnosis.

Patient-reported postoperative recovery and quality of life across the follow-up period were the principal outcomes of interest; no single primary assessment time point was prespecified. Longitudinal analyses of PSP, OHIP-14, and OQLQ total scores were considered the principal analyses. Comparisons at individual follow-up time points, OQLQ domain-specific analyses, and the evaluation of the association between baseline STE and postoperative PSP were considered exploratory.

### 2.3. Data Collection Methods

Patient-reported outcomes were collected electronically using Research Electronic Data Capture (REDCap; Vanderbilt University, Nashville, TN, USA). Assessments were performed preoperatively and at 24 h, 1, 3, and 6 weeks after surgery. The Short-Term Expectations (STE) questionnaire was administered only before surgery, to assess patients’ expectations regarding postoperative recovery. The Orthognathic Quality of Life Questionnaire (OQLQ) and the Oral Health Impact Profile-14 (OHIP-14) were administered at the preoperative assessment and at each subsequent follow-up visit, whereas the Postsurgical Perceptions (PSP) questionnaire was administered only during postoperative follow-up.

(T0) Before surgery—STE, OQLQ, OHIP-14.

(T1) 24-h post-surgery—OQLQ, OHIP-14, PSP.

(T2) 1-week post-surgery—OQLQ, OHIP-14, PSP.

(T3) 3-week post-surgery—OQLQ, OHIP-14, PSP.

(T4) 6-week post-surgery—OQLQ, OHIP-14, PSP.

### 2.4. Statistical Analysis

Continuous variables were summarized as means and standard deviations, and categorical variables as frequencies and percentages. Baseline comparisons between groups used the Welch t test (continuous variables) and the Fisher exact test (categorical variables). All tests were 2-sided, with significance set at *p* < 0.05.

The longitudinal evolution of PSP, OHIP-14, and OQLQ (total score and its 4 domains) were evaluated using linear mixed-effects models with a random intercept per participant, time modeled categorically, and fixed effects for surgical group, time, group × time, age, and dentofacial diagnosis. Models were estimated by restricted maximum likelihood, with type III tests (Satterthwaite approximation) for fixed effects and Holm-adjusted, model-derived estimated marginal means (95% CI) for between-group comparisons at each time point. All participants completed the scheduled follow-up assessments. OQLQ items answered as “not applicable” were treated as missing and were not assigned a score of 0. The primary analysis used available questionnaire data without imputation, and a sensitivity analysis used prorated OQLQ scores for questionnaires containing up to 4 missing or not-applicable items.

An exploratory model tested whether baseline STE (standardized) predicted postoperative PSP scores, adjusted for group, time, group × time, and age. Because the STE × time interaction was not significant (*p* = 0.180), a reduced model retained STE as a main effect; marginal PSP means were then estimated at −1 SD, mean, and +1 SD baseline STE.

Model assumptions (residual and quantile–quantile plots, singularity) and influential observations (standardized residuals, Cook’s distance) were assessed; models were re-estimated after excluding observations with standardized residuals >3 as a sensitivity analysis.

Interpretation relied on effect estimates and 95% confidence intervals, consistency between primary and sensitivity analyses, and the clinical trajectory of scores, rather than *p* values alone. Analyses were performed in R using RStudio, version 2024.04.2 (Posit Software, PBC, Boston, MA, USA); tables were formatted in Microsoft Excel (Microsoft Corporation, Redmond, WA, USA). As a pragmatic single-center cohort study, no a priori sample size calculation was performed; the analytic sample comprised all consecutive eligible patients treated during the enrollment period.

### 2.5. Surgical Technique

The bimaxillary orthognathic surgery for both groups was planned in the standard fashion according to the procedure typically performed by each surgeon, either COS or MIOS. When the patient agreed upon the treatment plan for bimaxillary orthognathic surgery, cone-beam computed tomography (CBCT) and intraoral dental scans were obtained, and a virtual surgical planning session were performed in conjunction with biomedical engineers. All surgeries were planned with patient-specific implants (PSI) using custom-made cutting guides and plates to stabilize the Le Fort I maxillary osteotomy, and conventional stock plates were used for the bilateral sagittal split osteotomy (BSSO) of the mandible. All patients underwent the same surgical sequence starting with making the BSSO corticotomies without completing the osteotomies, maxillary surgery to completion using either COS or MIOS approach to perform maxillary osteotomy and fixation, completion of the BSSOs of the mandible and final fixation of the mandibular segments. All patients were kept in the hospital for one night after surgery and discharged the following day once meeting postsurgical milestones. Postoperative pain regimen was the same for all patients.

The main difference between the two surgical procedures (COS/MIOS) was in the surgical approaches, i.e., the extent of the incisions, the amount of subperiosteal dissection, etc. (Table 1).

## 3. Results

A total of 41 patients met the inclusion criteria and completed the study (20 COS and 21 MIOS), with no losses to follow-up. Mean age, prevalence of anterior open bite, facial asymmetry, and baseline STE, OQLQ, and OHIP-14 scores did not differ significantly between groups (Table 2). The distribution of dentofacial diagnoses differed significantly (*p* = 0.013), with a greater proportion of Class II malocclusions in the MIOS group.

**Figure 1:**
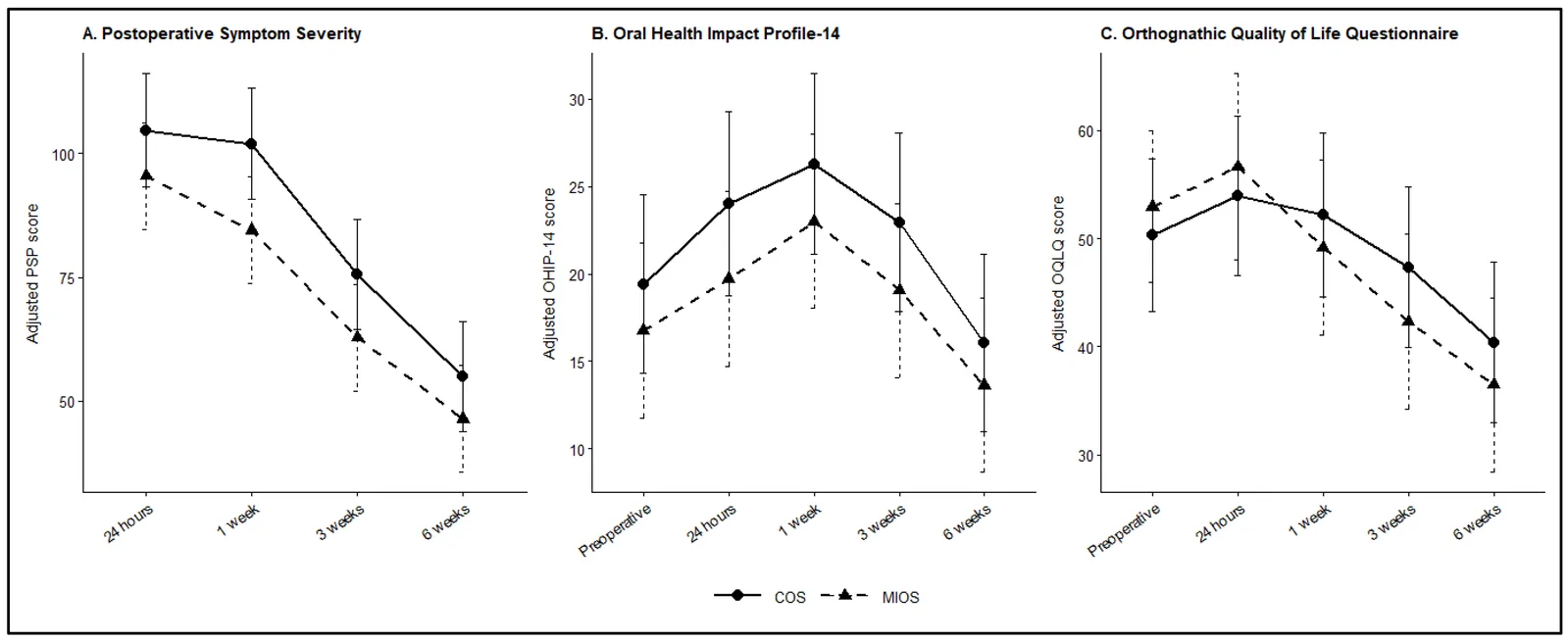
Patient-reported outcomes over time according to surgical approach: (**A**) PSP, (**B**) OHIP-14, and (**C**) OQLQ.

PSP scores decreased progressively throughout follow-up in both groups, with the highest scores observed at 24 h and the lowest at 6 weeks (Table 3, Figure 1A). The largest between-group difference in the observed data occurred at 1 week but did not reach statistical significance (*p* = 0.082). After adjustment for age and dentofacial diagnosis, PSP scores changed significantly over time (*p* < 0.001).

**Figure 2:**
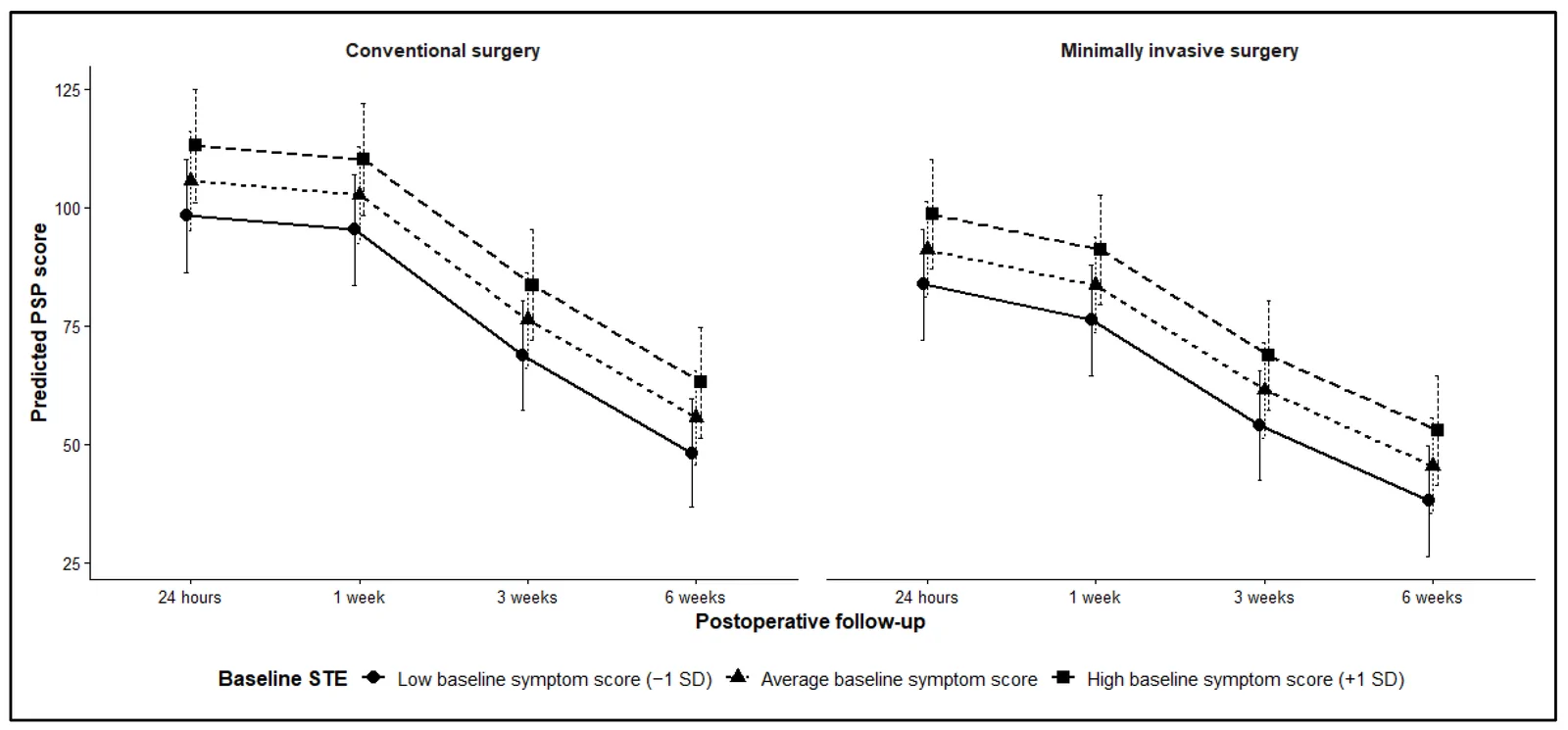
Predicted PSP trajectories according to baseline STE scores and surgical approach.

An exploratory mixed-effects model evaluated the association between baseline STE scores and postoperative PSP. Because the STE × time interaction was not significant (*p* = 0.180), a reduced model including STE as a main effect was used. After adjustment for age, surgical group, time, and the group × time interaction, higher baseline STE scores were independently associated with higher PSP scores throughout follow-up (β = 7.40; SE = 2.87; *p* = 0.014). Each 1-standard deviation increase in baseline STE was associated with an average 7.4-point increase in PSP. Time remained significantly associated with declining PSP scores (*p* < 0.001), whereas the group × time interaction remained non-significant. Predicted PSP trajectories according to low (−1 SD), average, and high (+1 SD) baseline STE scores are shown in Figure 2.

OHIP-14 scores increased during the early postoperative period and subsequently declined by the 6-week follow-up in both groups (Table 3, Figure 1B). After adjustment for age and dentofacial diagnosis, scores changed significantly over time (*p* < 0.001).

Similarly, OQLQ scores increased immediately after surgery and then progressively declined throughout follow-up in both groups (Table 2, Figure 1C). After adjustment for age and dentofacial diagnosis, scores changed significantly over time (*p* < 0.001).

**Figure 3:**
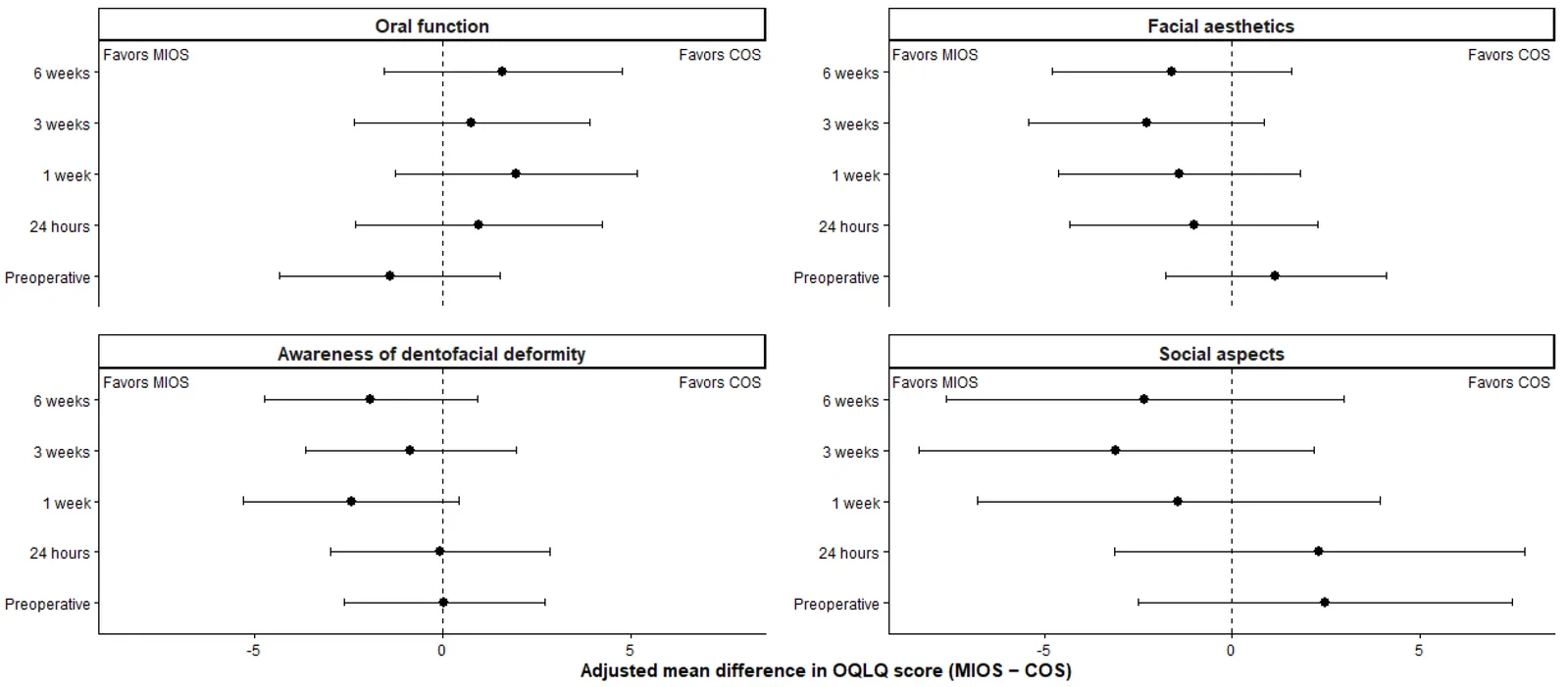
Adjusted differences in OQLQ domain scores between MIOS and COS throughout follow-up.

Across all four OQLQ domains, no statistically significant between-group differences were observed at any follow-up time point (Figure 3). The largest adjusted mean difference was observed for awareness of dentofacial deformity at 1 week, with MIOS patients scoring 2.44 points lower than COS patients (95% CI, −5.30 to 0.42; *p* = 0.093).

## 4. Discussion

Despite considerable heterogeneity in study design and outcome assessment, the literature consistently demonstrates improvement in Oral Health-Related Quality of Life (OHRQoL) following orthognathic surgery [6,13,14,15]. Improvements are particularly evident in facial esthetics and social or psychological well-being, although meaningful functional improvements also occur [6,15]. Prospective data similarly demonstrate substantial improvements in OQLQ and OHIP-14 scores by approximately 3–7 months after surgery [7,16], while Class III and bimaxillary patients may experience greater improvement in some domains than Class II or single-jaw patients [6,15,17].

Importantly, these long-term benefits are preceded by a period in which QoL may temporarily worsen. Conventional presurgical orthodontic decompensation can negatively affect facial appearance and OHRQoL, while the immediate postoperative period introduces pain, edema, dietary and functional limitations, altered sensation, and adaptation to a new occlusion. Consequently, early postoperative function and QoL may deteriorate before progressively improving with recovery [17,18]. This distinction between the eventual benefit of orthognathic surgery and the burden of recovering from it is clinically important. Although overall satisfaction following orthognathic surgery is high, dissatisfaction has been associated with postoperative complications, pain, hypoesthesia, prolonged treatment and recovery, inadequate preoperative information, and unmet expectations [14,19,20,21].

Currently, much of the existing literature evaluates QoL several months after surgery, when acute surgical morbidity has largely resolved. Studies remain heterogeneous in patient populations, procedures, instruments, and assessment intervals, and are predominantly observational with limited control of potential confounders [13,15,20]. Consequently, while the ultimate QoL benefit of orthognathic surgery is well established, considerably less is known about whether reducing the invasiveness of the surgical procedure itself can improve the early patient experience.

Our study builds upon this literature by evaluating whether the established postoperative QoL trajectory following conventional orthognathic surgery is altered by a minimally invasive surgical approach. Overall, both MIOS and COS demonstrated a recovery pattern consistent with that previously described following conventional orthognathic surgery. OHIP-14 and OQLQ scores initially worsened during the immediate postoperative period before progressively improving, with scores in both groups falling below their respective preoperative values by 6 weeks. This pattern is consistent with previous studies demonstrating transient deterioration in OHRQoL during early recovery followed by progressive improvement as patients adapt to their new occlusion and postoperative functional limitations resolve [17,18]. Although much of the existing literature has demonstrated these improvements at 3 to 7 months or later [7,16], the present findings suggest that the transition from acute postoperative deterioration toward improved QoL is already evident within the first several weeks following surgery.

Despite similar overall QoL trajectories, the findings suggest a possible difference in the early recovery experience between surgical approaches. PSP scores decreased significantly over time in both groups but were numerically lower following MIOS at every postoperative assessment. The greatest observed difference occurred at 1 week, when mean PSP scores were 84.5 following MIOS compared with 102.5 following COS (*p* = 0.082). Although these differences did not reach statistical significance, their direction and timing are noteworthy. The principal distinction between the surgical approaches was the extent of incision and subperiosteal dissection, while both groups underwent bimaxillary surgery using similar virtual planning, maxillary patient-specific implants, operative sequencing, hospitalization, and postoperative analgesic regimens. If limiting surgical exposure reduces patient-perceived morbidity, its greatest effect would be expected during the early postoperative period, when edema, pain, dietary limitations, and functional impairment are most pronounced.

The apparent difference between PSP and the broader QoL instruments may also reflect the distinct constructs measured by these questionnaires. The OQLQ and OHIP-14 principally measure the impact of the underlying dentofacial condition and oral health on patients’ functional, psychological, esthetic, and social well-being, whereas the PSP specifically assesses discomfort and difficulty experienced during postsurgical recovery. Because MIOS and COS ultimately address the same underlying dentofacial deformity, substantial differences in the eventual QoL benefit of skeletal correction would not necessarily be expected. Rather, the theoretical advantage of MIOS is a reduction in the morbidity required to achieve that correction. The consistent numerical separation in PSP scores, despite similar OQLQ and OHIP-14 trajectories, may therefore suggest that recovery-specific measures are more sensitive than general OHRQoL instruments for identifying potential patient-perceived differences attributable to surgical invasiveness. However, this interpretation remains exploratory given the absence of statistically significant between-group differences.

An additional finding was the relationship between preoperative expectations and postoperative recovery. Each 1-standard-deviation increase in baseline STE was independently associated with a 7.4-point increase in postoperative PSP scores (*p* = 0.014), and this association persisted throughout follow-up regardless of surgical approach. Although baseline STE scores did not differ significantly between the MIOS and COS groups, suggesting that expectations were unlikely to account for the observed between-group differences, these findings demonstrate that preoperative expectations independently influence perceived recovery burden. This finding is consistent with previous orthognathic literature identifying preoperative expectations, anticipated postoperative discomfort, psychological factors, and the quality of preoperative information as important determinants of postoperative perception and satisfaction [19,20,21]. Importantly, the present study prospectively measured anticipated short-term difficulty before surgery and demonstrated an association with subsequently reported recovery burden. These findings reinforce the importance of realistic preoperative counseling and suggest that the patient’s perception of recovery may be influenced not only by surgical morbidity but also by expectations entering the postoperative period.

The distribution of dentofacial deformities should also be considered when interpreting these findings. The MIOS cohort contained a greater proportion of Class II patients, whereas the COS cohort contained proportionally more Class III and other deformities (*p* = 0.013). Previous studies have suggested that Class III patients may experience greater improvements in OHRQoL than Class II patients following orthognathic surgery [7,15,16], although systematic evidence has not been entirely consistent [17]. Dentofacial diagnosis was therefore included as a covariate in the longitudinal models.

Several limitations should be considered when interpreting these findings. The relatively small sample size limited the ability to detect modest between-group differences. In addition, the nonrandomized design resulted in baseline differences in dentofacial diagnosis, and residual confounding may remain despite statistical adjustment. Surgical approach was also confounded by operating surgeon, as all MIOS procedures were performed by one surgeon and all COS procedures by another; therefore, differences between cohorts cannot be attributed solely to surgical technique and may also reflect surgeon-specific factors. Finally, objective measures of surgical morbidity, including operative time, blood loss, edema, analgesic consumption, neurosensory disturbances, and complications, were not prospectively evaluated because the study was designed specifically to assess patient-reported recovery and quality of life. Future prospective studies with larger samples, multiple surgeons performing both approaches, and complementary objective measures of surgical morbidity are warranted.

Taken together, the present findings suggest that MIOS does not fundamentally alter the early QoL trajectory associated with correction of dentofacial deformity. Rather, both approaches demonstrate the expected initial postoperative deterioration followed by progressive recovery. The potential advantage of MIOS may instead lie in reducing the patient-perceived burden during that recovery, as suggested by consistently lower PSP scores and the greatest numerical separation during the first postoperative week. These findings should not be interpreted as demonstrating superiority of MIOS, as no statistically significant differences in recovery trajectories were identified. Instead, they provide an early signal that reducing surgical exposure may influence recovery-specific patient-reported outcomes and support larger prospective studies specifically powered to evaluate differences during the acute postoperative period.

## 5. Conclusions

Although differences between-group were not statistically significant, postoperative symptom scores were numerically lower in the MIOS cohort throughout follow-up, with the greatest difference observed during early recovery. Larger studies are needed to determine whether these observed differences represent clinically meaningful patient-reported benefits of MIOS.

## Figures and Tables

**Table 1:** Main differences between COS and MIOS.

Conventional Orthognathic Surgery	Minimally Invasive Orthognathic Surgery
Incision made with electrocautery unit	Incision performed with a scalpel.
Maxillary incision extended from first molar to first molar	Maxillary incision made from lateral incisor to lateral incisor
Subperiosteal exposure of maxilla, zygoma and infraorbital rim	Subperiosteal exposure of inferior portion of maxilla only
Nasal muscles stripped from anterior nasal spine	Nasal muscles preserved on anterior nasal spine
Mandibular incision from mid-ramus to second premolar	Mandibular incision from mid-ramus to second molar
Subperiosteal exposure of mandible from second premolar to mid-ramus	Subperiosteal exposure of mandible from second molar to mid-ramus
BSSO from behind the lingula, down the ascending ramus, and mesial to the second molar area and vertically inferiorly to inferior border	BSSO from behind the lingula, down the ascending ramus, to the mesial of second molar, and then obliquely inferiorly toward the anterior portion of the mandibular angle
Maxillary incision closed with alar cinch and V-Y mucosal closure	Anterior nasal spine reconnected to maxilla. Maxillary incision closed without alar cinch or V-Y mucosal closure
Face tape not used for maxilla	Face tape used for maxilla

**Table 2:** Baseline characteristics of patients undergoing conventional or minimally invasive orthognathic surgery.

Characteristic	COS (n = 20)	MIOS (n = 21)	*p* Value
**Age, years**	25.9 ± 10.1	27.3 ± 13.2	0.708
**Dentofacial diagnosis, n (%)**			0.013
**Class II**	3 (15.0)	10 (47.6)	
**Class III**	12 (60.0)	11 (52.4)	
**Other or not classifiable**	5 (25.0)	0	
**Open bite, n (%)**	4 (20.0)	3 (14.3)	0.697
**Facial asymmetry, n (%)**	3 (15.0)	9 (42.9)	0.085
**Baseline patient-reported outcome measures**			
**STE score**	67.0 ± 19.5	69.1 ± 28.3	0.791
**OQLQ score**	49.9 ± 17.5	52.9 ± 16.8	0.591
**OHIP-14 score**	19.4 ± 12.3	16.8 ± 11.7	0.485

Abbreviations: COS, conventional orthognathic surgery; MIOS, minimally invasive orthognathic surgery; OHIP-14, Oral Health Impact Profile-14; OQLQ, Orthognathic Quality of Life Questionnaire; STE, Short-Term Expectations. Continuous variables are presented as mean ± standard deviation and categorical variables as number (percentage).

**Table 3:** Observed patient-reported outcome scores according to surgical group and follow-up.

Outcome	Follow Up	COS	MIOS	*p* Value
**PSP**	**24 h**	103.3 ± 28.1	95.5 ± 27.2	0.436
	**1 week**	102.5 ± 30.1	84.5 ± 26.2	0.082
	**3 weeks**	75.6 ± 27.9	62.8 ± 16.6	0.24
	**6 weeks**	55.0 ± 24.1	46.3 ± 16.4	0.501
**OHIP-14**	**Preoperative**	19.4 ± 12.3	16.8 ± 11.7	0.635
	**24 h**	23.1 ± 14.1	19.7 ± 9.7	0.369
	**1 week**	26.3 ± 13.5	23.0 ± 14.1	0.524
	**3 weeks**	23.0 ± 10.5	19.1 ± 10.2	0.428
	**6 weeks**	16.1 ± 9.4	13.6 ± 8.3	0.672
**OQLQ**	**Preoperative**	49.9 ± 17.5	52.9 ± 16.8	0.696
	**24 h**	53.1 ± 15.4	58.0 ± 19.4	0.736
	**1 week**	50.7 ± 16.7	49.5 ± 15.1	0.58
	**3 weeks**	46.0 ± 14.3	41.3 ± 11.2	0.364
	**6 weeks**	39.2 ± 15.0	37.2 ± 11.2	0.48

Abbreviations: COS, conventional orthognathic surgery; MIOS, minimally invasive orthognathic surgery; OHIP-14, Oral Health Impact Profile-14; OQLQ, Orthognathic Quality of Life Questionnaire; PSP, Postsurgical Perceptions. Values are presented as mean ± standard deviation at each assessment time using the observed data available for each outcome.

## Data Availability

The data presented in this study are available on request from the corresponding author. The data are not publicly available due to patient privacy and confidentiality restrictions.

## Abbreviations

The following abbreviations are used in this manuscript:

BSSO	Bilateral sagittal split osteotomy
CBCT	Cone beam computed tomography
CI	Confidence interval
COS	Conventional orthognathic surgery
MIOS	Minimally invasive orthognathic surgery
OHIP-14	Oral Health Impact Profile-14
OHRQoL	Oral health-related quality of life
OQLQ	Orthognathic Quality of Life Questionnaire
PSI	Patient-specific implant
PSP	Postsurgical Perceptions
QoL	Quality of life
REDCap	Research Electronic Data Capture
SD	Standard deviation
STE	Short-Term Expectations
